# Store-Operated Calcium Entry Contributes to Cisplatin-Induced Cell Death in Non-Small Cell Lung Carcinoma

**DOI:** 10.3390/cancers11030430

**Published:** 2019-03-26

**Authors:** Roberta Gualdani, Marie de Clippele, Ikram Ratbi, Philippe Gailly, Nicolas Tajeddine

**Affiliations:** Laboratory of Cell Physiology, Institute of Neuroscience, Université Catholique de Louvain, Brussels 1200, Belgium; roberta.gualdani@uclouvain.be (R.G.); marie.declippele@uclouvain.be (M.d.C.); ikram.ratbi@uclouvain.be (I.R.); philippe.gailly@uclouvain.be (P.G.)

**Keywords:** store-operated calcium entry, cisplatin, apoptosis, reactive oxygen species, mitochondrial calcium, non-small cell lung carcinoma

## Abstract

Cisplatin (CDDP) is one of the principal chemotherapeutic agents used for the first-line treatment of many malignancies, including non-small cell lung carcinoma (NSCLC). Despite its use for over 40 years, its mechanism of action is not yet fully understood. Store-operated calcium entry (SOCE), the main pathway allowing Ca^2+^ entry in non-excitable cells, is involved in tumorogenesis, cancer progression and chemoresistance. It has become an attractive target in cancer treatment. In this study, we showed that siRNA-mediated depletion of stromal interaction molecule 1 (STIM1) and transient receptor potential channel 1 (TRPC1), two players of the store-operated calcium entry, dramatically reduced CDDP cytotoxicity in NSCLC cells. This was associated with an inhibition of the DNA damage response (DDR) triggered by CDDP. Moreover, STIM1 depletion also reduced CDDP-dependent oxidative stress. In parallel, SOCE activation induced Ca^2+^ entry into the mitochondria, a major source of reactive oxygen species (ROS) within the cell. This effect was highly decreased in STIM1-depleted cells. We then conclude that mitochondrial Ca^2+^ peak associated to the SOCE contributes to CDDP-induced ROS production, DDR and subsequent apoptosis. To the best of our knowledge, this is the first time that it is shown that Ca^2+^ signalling constitutes an initial step in CDDP-induced apoptosis.

## 1. Introduction

The anticancer activity of cisplatin (*cis*-diamminedichloroplatinum(II), CDDP) was shown for the first time by Barnett Rosenberg in 1969 [1]. It was FDA-approved for treatment of testicular and ovarian cancers in 1978. However, despite its long-standing and large use in the treatment of many malignancies, including sarcomas, carcinomas of lung, ovaries, head and neck and bladder, its mechanism of action is not yet completely understood [2]. The “canonical” pathway underlying the anticancer effects of CDDP is linked to its ability to form intrastrand and interstrand DNA-adducts and therefore to trigger DNA damage response (DDR) pathway. This leads to p53 accumulation and mitochondrial membrane permeabilization (MMP), which is the rate-limiting step of the intrinsec pathway of apoptosis [3]. However, intriguing results showed that CDDP induced mitochondrial-dependent apoptosis in enucleated cells, demonstrating that DNA is dispensable for CDDP-induced cell death [4,5]. This may be related to direct prooxidant activity of CDDP and/or to a reduction of antioxidant capacity of the cell. Moreover, oxidative stress triggers DDR and reactive oxygen species (ROS) might then constitute an intermediate signal between CDDP and DNA [6]. Other mechanisms have been suggested to explain the anti-cancer activity of CDDP. Among them, it has been shown that CDDP induced ERK activation. However, it is not clear whether this effect is involved in CDDP-induced cell death or, on the contrary, mediates resistance against CDDP cytotoxicity [7,8,9,10].

A myriad of studies show that alterations in Ca^2+^ signalling initiate or support the development of hallmarks of cancer (for an extensive review, see [11]). Ca^2+^ homeostasis is also involved in chemoresistance [12]. For instance, Ca^2+^ might modulate expression of multidrug resistance (MDR) genes [13]. The increased Ca^2+^ entry through the upregulated ion channel TRPC5, besides its effects on the expression of MDR genes, allows the activation of autophagy that protects cancer cells against cell death [14]. Furthermore, in breast cancer, TRPC5 may be transferred from resistant cancer cells to sensitive cancer cells via a vesicular shuttle [15]. Ca^2+^ release from the endoplasmic reticulum (ER) through the ryanodine receptor contributes to the acquisition of a stem cell-like phenotype that promotes chemoresistance [16].

Despite intensive researches focusing on the role of Ca^2+^ in tumorogenesis, cancer progression and chemoresistance, scarce data are available concerning Ca^2+^ signalling in CDDP-dependent cytotoxicity. CDDP triggers an entry of Ca^2+^ in HeLa-S3 but not in U2-OS cells [17]. Inhibition of this Ca^2+^ entry attenuates CDDP-induced cell death. In head and neck squamous cell carcinoma, phospholipase C and IP3R may contribute to CDDP-resistance [18]. In ovarian cancer cell lines, CDDP induces a release of Ca^2+^ from the stores, which activates calpains that in turn degrade p73. This mechanism was abolished in CDDP-resistant isogenic cell lines [19]. Finally, it has been reported that alterations in Ca^2+^ homeostasis contributed to CDDP-resistance in a breast cancer cell line [20].

Until now, the role of store-operated calcium entry (SOCE) in CDDP-induced cell death has been poorly investigated. And yet, SOCE is the main mechanism by which external Ca^2+^ enters into non-excitable cells. ER depletion induces the translocation of stromal interaction molecule 1 (STIM1), an ER membrane protein, to ER/PM junctional regions. STIM1 then opens ORAI1, a PM channel, which allows Ca^2+^ entry from the external medium [21,22,23]. In osteosarcomas, it has been shown that STIM1 upregulation was associated with a CDDP-resistant phenotype [24]. ORAI1 and STIM1 are anti-apoptotic in pancreatic adenocarcinoma cell lines and siRNA-mediated depletion of ORAI1 and STIM1 increased apoptosis induced by 5-fluorouracil (5-FU) or gemcitabine [25]. In HepG2 hepatocarcinoma cells, inhibition of Ca^2+^ entry through ORAI1 increased autophagic cell death induced by 5-FU [26].

In this study, we examined the effect of SOCE inhibition on CDDP-induced cell death in a model of non-small cell lung carcinoma (NSCLC). We observed that inhibition of SOCE dramatically decreased cytotoxicity induced by CDDP. The DDR pathway was also impaired after SOCE inhibition. Finally, production of ROS triggered by CDDP was lowered in the absence of SOCE. This effect could be mediated by a decrease of mitochondrial Ca^2+^ elevation after SOCE activation. We then suggest that SOCE is required to allow CDDP-dependent ROS production and the consecutive activation of the DDR pathway.

## 2. Results

### 2.1. SOCE Inhibition Reduced CDDP-Dependent Cell Death

**Figure 1:**
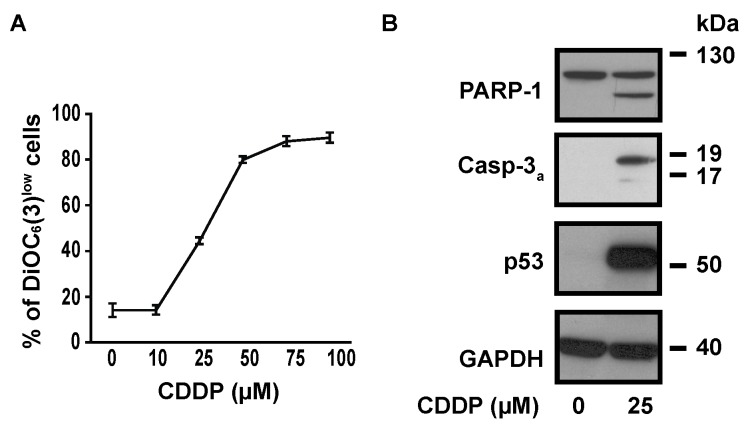
Dose-response relationship in CDDP-treated A549 cells. (**A**) Cytofluorometric assessment of ΔΨ_m_ (with the ΔΨ_m_-sensitive probe DiOC_6_(3)) after 24 h treatment with CDDP at indicated concentrations. (**B**) Immunoblot analysis showing the effect of 24 h treatment with 25 µM CDDP on PARP-1 cleavage, caspase-3 cleavage and p53 accumulation. GAPDH is used as a loading control.

To determine the optimal concentration of CDDP to induce cell death in NSCLC A549 cells, we measured CDDP-induced transmembrane mitochondrial potential (ΔΨm) dissipation with the ΔΨm-sensitive fluorochrome DiOC_6_(3). The dose response curve showed that CDDP EC_50_ was about 25 µM (Figure 1A). This concentration was then used for the majority of following experiments. 25 µM CDDP induced the cleavage of caspase-3 and of its substrate poly(ADP-ribose) polymerase 1 (PARP-1), indicating that it promotes apoptotic cell death at this concentration (Figure 1B). As expected, a strong accumulation of p53 was observed after 24 h treatment with 25 µM CDDP (Figure 1B).

**Figure 2:**
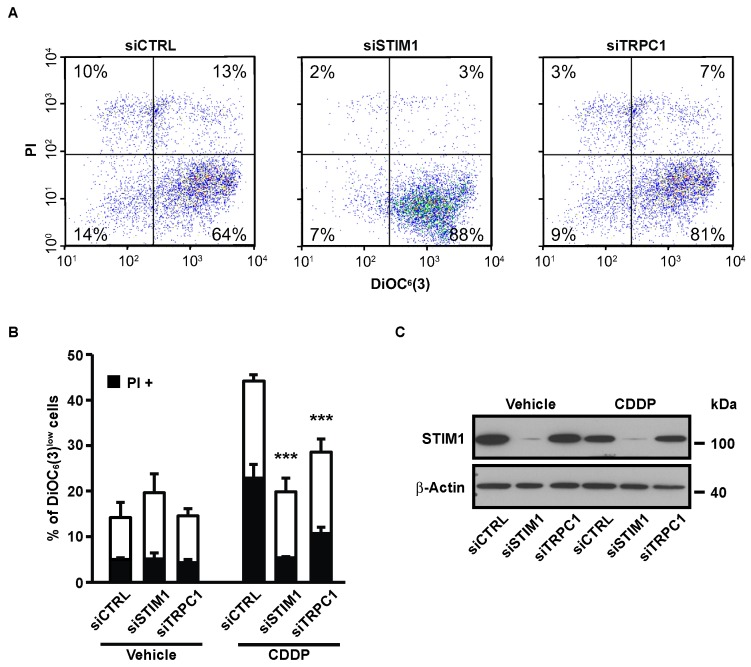
Effects of STIM1 and TRPC1 depletion on apoptosis-associated mitochondrial transmembrane potential (ΔΨ _m_) dissipation and plasma membrane permeabilization. (**A**) Following transient transfection (for 72 h) with either siRNA downregulating STIM1 (siSTIM1) or TRPC1 (siTRPC1) or with a control siRNA (siCTRL), A549 cells were left untreated or treated for additional 24 h with 25 µM CDDP, and labeled for the cytofluorometric assessment of ΔΨ_m_ (with the ΔΨ_m_-sensitive probe DiOC_6_(3)) and plasma membrane integrity (with propidium iodide, i.e., PI). (**B**) Quantification of data presented in A. White and grey columns represent the percentage of cells exhibiting ΔΨ_m_ loss alone (DiOC_6_(3)^low^) or in association with plasma membrane breakdown (PI^+^), respectively. Data are means of triplicate experiments ± S.D. and are representative of three independent experiments. Student’s *t* test was employed to assess statistical significance. *** *p* < 0.001. (**C**) Immunoblot analysis of STIM1 expression 96 h after transfection of A549 cells with siCTRL, siSTIM1 or siTRPC1. β-actin was used as loading control.

Depletion of STIM1 with a pool of four different siRNAs (named siSTIM1) reduced CDDP-induced ΔΨm dissipation and plasma membrane permeabilization (measured by the vital dye propidium iodide, PI) (Figure 2A,B). We previously showed that the cation channel TRPC1 mediated SOCE in A549 cells [27]. In this study, we observed that siRNA-mediated depletion of TRPC1 decreased CDDP-induced apoptosis in a similar level to STIM1 depletion (Figure 2A,B).

This confirms that the effect of STIM1 depletion on CDDP-induced apoptosis is not related to an off-target effect of the siRNA or to an unknown function of STIM1 but is actually due to SOCE inhibition. siRNA-mediated STIM1 depletion was confirmed by immunoblotting (Figure 2C). We previously showed the efficiency of the same siRNAs targeted against TRPC1 in A549 cells [27].

### 2.2. SOCE Inhibition Reduced Expression of Specific Markers of Apoptosis Induced by CDDP

**Figure 3:**
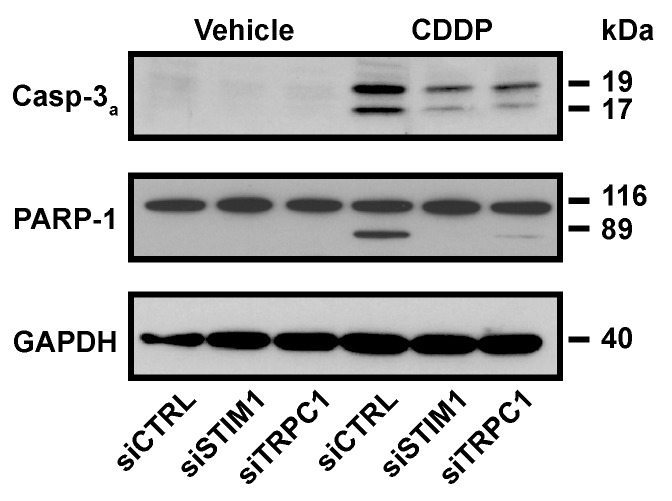
Influence of STIM1 and TRPC1 depletion on CDDP-induced apoptosis. Immunoblot analysis showing the effect of 24 h treatment with 25 µM CDDP on caspase-3 activation and PARP-1 cleavage in siCTRL, siSTIM1 or siTRPC1 transfected cells. GAPDH is used as a loading control. One result representative of at least three independent experiments.

SOCE inhibition reduced biochemical hallmarks of the apoptosis activated by CDDP. As expected, 19 kDa and 17 kDa fragments from caspase-3, that is the consequence of its activation, were detected after CDDP treatment. STIM1 and TRPC1 depletion decreased CDDP-induced expression of active fragments of caspase-3, and this was correlated with a reduced degradation of PARP-1 (Figure 3).

### 2.3. SOCE Was Not Altered by CDDP

**Figure 4:**
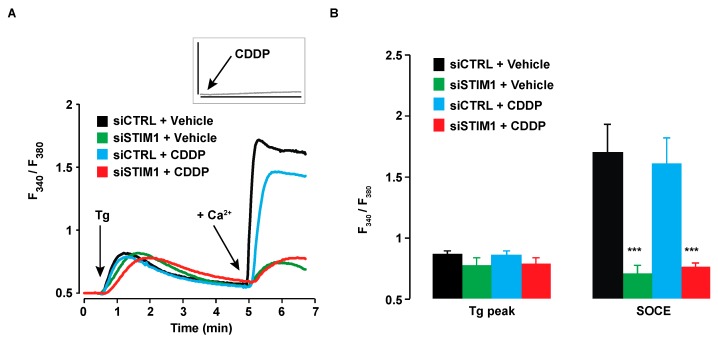
Influence of STIM1 depletion and CDDP on SOCE. (**A**) After transfection with siCTRL or siSTIM1, A549 cells were pretreated for 6 h with 25 µM CDDP or the vehicle and then stained with Fura-2/AM to allow cytosolic Ca^2+^ measurement. Tg was added in the absence of external Ca^2+^, in a solution of Krebs EGTA. The first Ca^2+^ peak reflects the release of Ca^2+^ from the ER. After ~ 4 min, Ca^2+^ was added in the external medium, allowing SOCE. Traces are representative of at least 5 independent experiments (minimum 10 cells analysed per experiment). The acute effect of 25 µM CDDP on cytosolic Ca^2+^ is shown in the insert (same scale as the main graph). (**B**) Quantification of experiments presented in A. Results are expressed as means ± S.D. (*n* ≥ 5). Student’s *t* test was employed to assess statistical significance. *** *p* < 0.001.

CDDP by itself was not able to induce Ca^2+^ transients in A549 cells (Figure 4A, insert). Moreover, CDDP did not modify the Ca^2+^ concentration within the ER, as reflected by the absence of effect of CDDP on cytosolic Ca^2+^ increase elicited by the SERCA pump inhibitor thapsigargin (Tg) in the absence of Ca^2+^ in the external medium (Figure 4A,B). To measure SOCE amplitude, we re-added Ca^2+^ in the external medium after Tg-induced ER emptying. As expected, SOCE amplitude was highly decreased in siSTIM1 transfected cells (Figure 4B). However, CDDP had no effect on SOCE amplitude.

### 2.4. STIM1 Depletion Inhibited CDDP-Dependent ERK Activation

**Figure 5:**
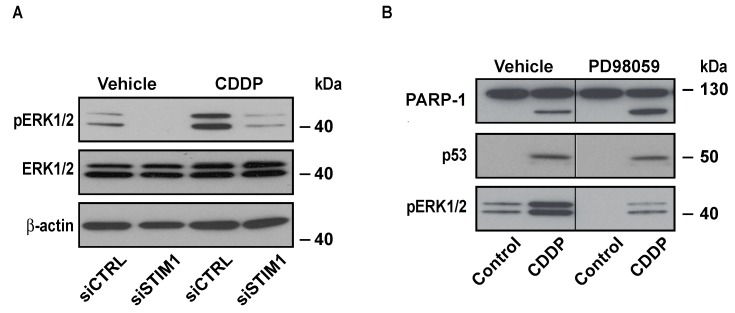
Effect of STIM1 depletion on ERK1/2 phosphorylation induced by CDDP. (**A**) Immunoblot analysis showing the effect of 24 h treatment with 25 µM CDDP on pERK1/2 in siCTRL or siSTIM1 transfected cells. (**B**) Immunoblot analysis showing the effect of 50 µM PD98059 of PARP-1 cleavage and p53 accumulation induced by 24 h treatment with 25 µM CDDP. Lanes separated by a thin line have been cropped from the same immunoblot.

As mentioned above, CDDP is known to induce ERK activation. We confirmed that, in A549 cells, 25 µM CDDP increased phosphorylation of ERK1/2. Depletion of STIM1 almost abolished this effect (Figure 5A). This is in line with our previous data showing that siTRPC1 inhibited ERK1/2 activation triggered by EGF in A549 cells [27]. Since the role of CDDP-dependent activation of ERK1/2 in cell death is a matter of debate, we inhibited the ERK1/2 pathway with PD98059 to identify a potential role of this pathway in apoptosis induced by CDDP. CDDP-induced PARP-1 cleavage was not modified by PD98059 (Figure 5B). This demonstrated that the effect of STIM1 depletion on CDDP-induced cell death might not be explained by an inhibition of the ERK1/2 pathway. In line with this observation, accumulation of p53 triggered by CDDP was not impaired in the presence of PD98059 (Figure 5B).

### 2.5. STIM1 Depletion Inhibited DDR Induced by CDDP

**Figure 6:**
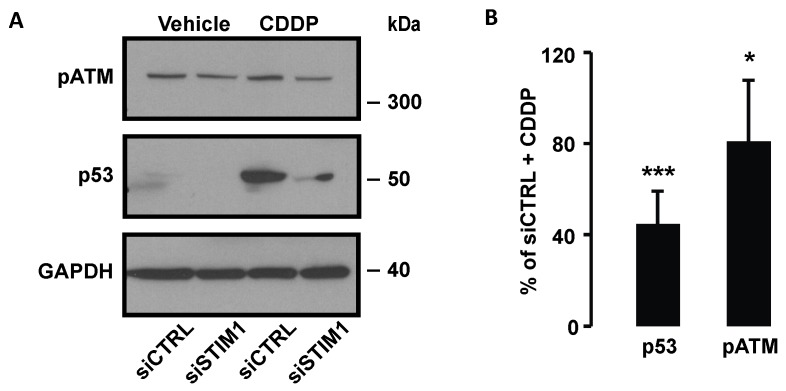
Effect of STIM1 depletion on CDDP-induced DDR. (**A**) Immunoblot analysis showing the effect of 24 h treatment with 25 µM CDDP on pATM and p53 level in siCTRL or siSTIM1 transfected cells. One immunoblot representative of at least three independent experiments. (**B**) Quantification of experiments presented in **A**. Results are expressed as means ± S.D. (*n* ≥ 3). Student’s *t* test was employed to assess statistical significance. * *p* < 0.05; *** *p* < 0.001.

It is well known that CDDP induces DDR by forming DNA-adducts or by producing ROS that in turn alter DNA. To detect activation of the DDR pathway, we measured phosphorylation of ATM, which is provoked by DNA lesions. As expected, CDDP induced ATM phosphorylation (Figure 6A). This effect was reduced after STIM1 depletion. In response to DDR activation, p53 accumulates within the cell and activates intrinsic pathway of apoptosis or cell growth arrest. CDDP-dependent p53 accumulation was dramatically decreased in STIM1-depleted cells (Figure 6A,B). This means that SOCE is required to trigger DDR after CDDP treatment. 

### 2.6. STIM1 Depletion Reduced CDDP-Induced ROS Production

**Figure 7:**
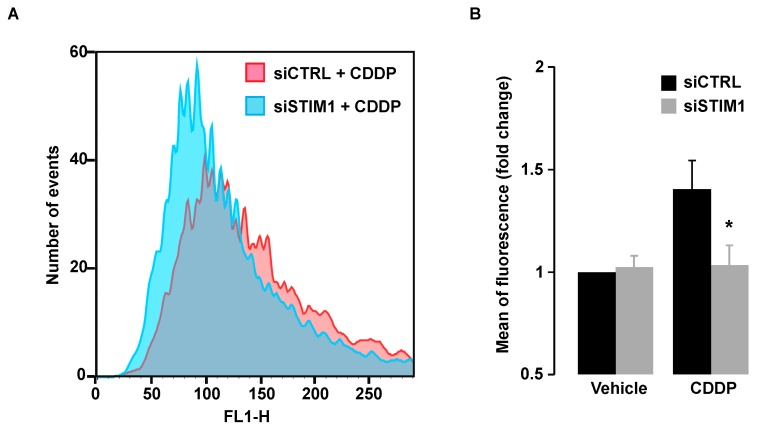
Cytofluorometric assessment of ROS generation. (**A**) Following transient transfection (for 72 h) with either siRNA downregulating STIM1 (siSTIM1) or with a control siRNA (siCTRL), A549 cells were left untreated or treated for additional 24 h with 25 µM CDDP, and labeled with H2DCFDA for ROS detection. (**B**) Quantification of data presented in **A**. Data are normalized to control conditions (i.e., siCTRL transfected cells without CDDP) and presented as the mean of three independent experiments ± S.D. ANOVA test was employed to assess statistical significance. * *p* < 0.05.

Since CDDP-triggered DDR was inhibited after SOCE inhibition, this suggested that DNA damage was not only induced by a direct effect of CDDP on DNA. As mentioned in the introduction, CDDP is known to elicit the production of ROS that might in turn damage DNA. In line with those observations, we showed that CDDP induced production of ROS that were measured by the increase of fluorescence of the H2DCFDA probe (Figure 7A,B). We observed that ROS production after CDDP exposure was abolished in STIM1-depleted cells (Figure 7A,B), indicating that production of ROS after CDDP exposure depends on Ca^2+^ entry through store-operated channels.

### 2.7. STIM1 Depletion Reduced SOCE-Dependent Entry of Ca^2+^ into the Mitochondria

**Figure 8:**
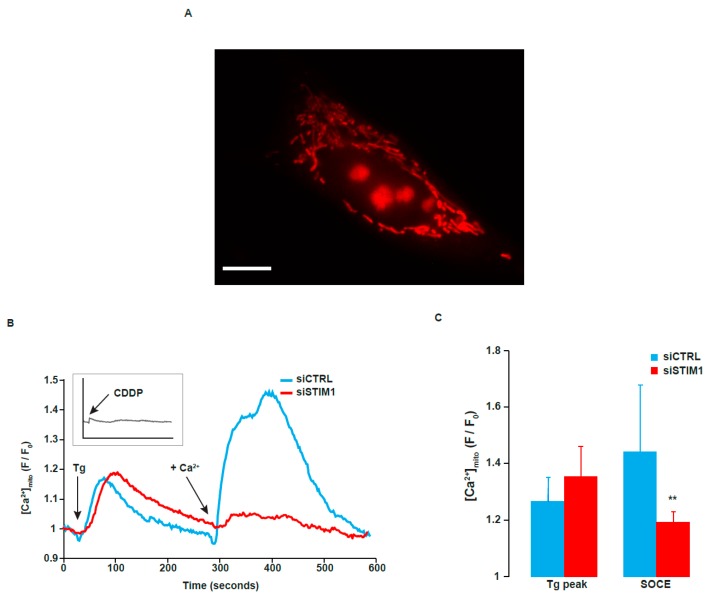
Influence of STIM1 depletion on mitochondrial Ca^2+^ concentration. (**A**) After transfection with siCTRL or siSTIM1, A549 cells were stained with Rhod-2/AM to allow cytosolic mitochondrial Ca^2+^ measurement. Tg was added in the absence of external Ca^2+^, in a solution of Krebs EGTA. The first Ca^2+^ peak reflects the release of Ca^2+^ from the ER. After ~4 min, Ca^2+^ was added in the external medium, allowing SOCE. A Representative picture of A549 cell stained with Rhod-2. Scale bar = 10 µm. (**B**) Traces are representative of at least eight independent experiments (between 5 and 20 cells analyzed per experiment). Fluorescence at the indicated time is related to the fluorescence at the beginning of the experiment (F/F_0_). The acute effect of 25 µM CDDP on mitochondrial Ca^2+^ is shown in the insert (same scale as the main graph). (**C**) Quantification of experiments presented in B. Results are expressed as means ± S.D. (*n* = 8 for siCTRL transfected cells and *n* = 9 for siSTIM1 transfected cells). Student’s *t* test was employed to assess statistical significance. ** *p* < 0.01.

Since mitochondria constitute a major source of ROS and that mitochondrial Ca^2+^ is involved is mitochondrial ROS production, we measured mitochondrial Ca^2+^ after activation of SOCE. To achieve that, we used the cell-permeant fluorescent Ca^2+^ indicator Rhod-2 AM. Fluorescent imaging of A549 cells stained with this dye exhibited a typical mitochondrial pattern (Figure 8A). Tg-induced release of Ca^2+^ from the ER induced a peak of Ca^2+^ within the mitochondrial matrix (Figure 8B,C). This effect was similar in control cells and in siSTIM1-transfected cells. In contrast, STIM1 depletion dramatically reduced mitochondrial Ca^2+^ peak after re-addition of Ca^2+^ in the external medium (Figure 8B,C). This is correlated with the decrease of SOCE-evoked cytosolic Ca^2+^ peak and with the decrease of ROS production in STIM1-depleted cells. As in the cytosol, CDDP by itself did not affect mitochondrial Ca^2+^ (Figure 8B, insert).

### 2.8. SOCE Is Also Involved in CDDP-Induced Cell Death in Cervix Carcinoma Cells

**Figure 9:**
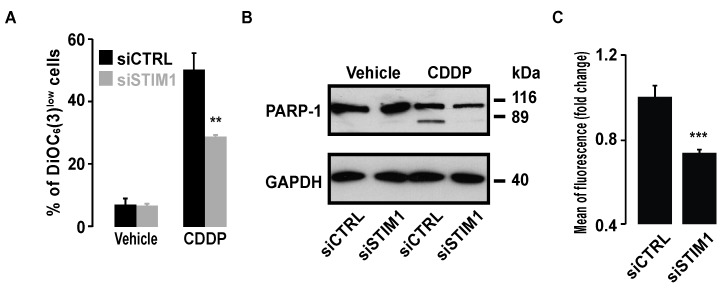
Effect of STIM1 depletion on CDDP-induced cell death and ROS production in HeLa cells. (**A**) Bar histogram showing the percentage of cells with low ΔΨ_m_ in the presence of 25 µM CDDP in siCTRL or siSTIM1 transfected HeLa cells. Data are means of three experiments ± S.D. Student’s *t* test was employed to assess statistical significance. ** *p* < 0.01. (**B**) Immunoblot analysis showing the effect of 24 h treatment with 25 µM CDDP on PARP-1 cleavage in siCTRL or siSTIM1 transfected cells. GAPDH was used as a loading control. (**C**) Bar histogram showing the mean level of fluorescence emitted by the H2DCFDA probe allowing ROS production assessment in the presence of 25 µM CDDP in siCTRL or siSTIM1 transfected HeLa cells. Data are means of three experiments ± S.D. Student’s *t* test was employed to assess statistical significance. *** *p* < 0.001.

We aimed to investigate whether the results that we obtained in NSCLC were also true in another cancer routinely treated by CDDP. We observed that STIM1 depletion also decreased CDDP-induced cell death in cervix carcinoma cells HeLa, as demonstrated by the reduction of the percentage of cells with low ΔΨm and the reduction in PARP cleavage (Figure 9A,B).

This last result demonstrates that caspase activation in response to CDDP was inhibited in the absence of STIM1. As in A549 cells, ROS production in response to CDDP was also decreased in STIM1-depleted cells (Figure 9C).

## 3. Discussion

Several studies suggest that SOCE components might be attractive targets in cancer treatment. In NSCLC, we showed that SOCE ablation reduced proliferation rate by disrupting EGFR-dependent signalling [27]. More recently, we discovered that disruption of the EGFR/ErbB2-dependent signalling by lapatinib and CP-724714, two inhibitors of the receptor tyrosine kinase (RTK), diminished the amplitude of the SOCE in breast cancer cells [28]. In the present study, we showed that siRNA-mediated SOCE inhibition dramatically reduced CDDP cytotoxicity in NSCLC. Interestingly, a similar effect was obtained in the cervix carcinoma cell line HeLa, suggesting that our observations are not restricted to a single type of cancer. Surprisingly, a previous report showed a slight increase in CDDP-induced apoptosis after STIM1 silencing [29]. We must admit that we do not have any convincing arguments to explain this discrepancy. The decreased cytoxicity after STIM1 depletion was correlated with a drop of oxidative stress triggered by CDDP and with a reduction of mitochondrial Ca^2+^ peak following SOCE. This observation was in line with our previous results in skeletal muscles [30]. In contrast, CDDP was not able to induce Ca^2+^ transient or to alter SOCE. A potential role of the MAPK pathway in CDDP-induced apoptosis has previously been described. We actually observed a major increase in the expression of pERK1/2 in A549 cells after CDDP exposure, effect that was considerably reduced in STIM1-depleted cells. However, pharmacological inhibition of ERK1/2 did not alter the potency of CDDP to induce cell death. We conclude that the MAPK pathway does not play a significant role in CDDP-induced apoptosis in A549 cells.

Production of ROS after CDDP treatment is well documented. This oxidative stress can contribute to the anti-tumor activity of CDDP [31,32,33]. Unfortunately, the well-known toxicity of CDDP, especially on kidney, is also related to ROS production [34]. The production of ROS by CDDP treatment mainly depends on the depletion of antioxidant molecule like glutathione and on injury in the mitochondrial respiratory chain [35].

Ca^2+^ signalling can elicit the production of ROS, essentially via direct activation NADPH oxidase isoforms or by increasing the activity of Krebs cycle and electron transport chain enzymes that in turn drive the production of superoxide [36]. Reciprocally, ROS regulate Ca^2+^ signalling at multiple levels and increasing evidences show that this crosstalk between Ca^2+^ and ROS is of significant importance in tumorogenesis (for a recent and extensive review on this topic, see [37]).

More specifically, ROS are also known to regulate SOCE. Indeed, oxidation of cysteine 49 and 56 of STIM1 inhibits STIM1 oligomerization and SOCE [38]. Similarly, oxidation of ORAI1 locks the channel in a closed conformation [39]. However, much less studies have investigated the control of ROS production by SOCE. Here, we show for the first time that SOCE inhibition is able to reduce CDDP-induced ROS production. The mechanism underlying this effect is not clear. Mitochondria might constitute a possible target of STIM1-dependent Ca^2+^ entry. Since Ca^2+^ stimulates several enzymes of the Krebs cycle, the increased metabolic rate induced by Ca^2+^ overload within the mitochondria might result in increased respiratory chain leakage and ROS levels [35]. Moreover, it has been shown that SOCE inhibition reduced oxidative stress in a cellular model of Parkinson’s disease in which oxidative phosphorylation in mitochondria are injured by 1-methyl-4-phenylpyridinium (MPP+) [40].

## 4. Materials and Methods

### 4.1. Cell Culture and Reagents

A549 and HeLa cell lines (American Type Culture Collection, Molsheim, France) were grown in DMEM-F12 and in DMEM, respectively, supplemented with 10% FCS at 37 °C in a humidified atmosphere of 5% CO_2_. Cells were cultured up to passage 30. CDDP, thapsigargin and PD98059 were purchased from Sigma-Aldrich (Overijse, Belgium).

### 4.2. siRNA Transfection

Depletion of STIM1 and TRPC1 was achieved by using a pool of four siRNAs (called siSTIM1 and siTRPC1, respectively) targeting four different sequences of human STIM1 and TRPC1 mRNA. siSTIM1 (Catalog #L-011785-00), siTRPC1 (Catalog #L-004191-00-0005) as well as the non-silencing control pool of siRNAs (siUNR, Catalog #D-001810-10) were purchased from Dharmacon (Cambridge, UK). A549 cells were transfected using DharmaFECT reagent according to the manufacturer’s instructions (Dharmacon). Cells were analyzed 96 h after transfection.

### 4.3. Immunoblotting

A549 cells were harvested by scraping in PBS, rinsed twice with ice-cold PBS, and then re-suspended in lysis buffer (Cell Lysis Buffer, Abcam, Cambridge, UK) containing 0.216% β-glycerophosphate, 0.19% sodium orthovanadate, 0.001% leupeptin, 0.38% EGTA, 10% Triton X-100, 3.15% Tris HCl, 8.8% Sodium chloride, 0.29% sodium EDTA and 1.12% sodium pyrophosphate decahydrate.

Extracts were then diluted in a mix of LDS sample buffer and sample reducing Agent (NuPAGE^®^, Fisher Scientific, Erembodegem, Belgium) and heated at 95 °C for 3 min. Samples were electrophoresed on 7% or 10% SDS-polyacrylamide gels (Invitrogen) and transferred onto nitrocellulose (Bio-Rad, Temse, Belgium) or PVDF membranes (Merck Millipore, Overijse, Belgium). The blots were saturated with TBS-T buffer (0.02 M Tris(hydroxymethyl)aminomethane, 0.138 M NaCl, 0.05% Tween 20, pH 7.6), containing 5% BSA or milk for 1 h at room temperature, incubated overnight at 4 °C with primary antibodies: anti-STIM1, anti-cleaved caspase-3, anti-PARP-1, anti-p53, anti-p44/42 MAPK, anti-phospho-p44/42 MAPK, anti-GAPDH (all of them diluted at 1:1000 and provided by Cell Signaling Technology, Leiden, The Netherlands) and anti-β-actin (1:10,000, Sigma-Aldrich). After incubation with appropriate secondary antibodies coupled to peroxidase, peroxidase activity was detected with ECL (Westar C2.0, Cyanagen, Bologna, Italy) on ECL hyperfilm. Immunoblots were quantified using the ImageJ program (National Institute for Health, Bethesda, MA, USA). All lanes separated by a thin line have been cropped from the same immunoblot.

### 4.4. Cytosolic and Mitochondrial Free Ca^2+^ Measurements

A549 cells were plated on 22-mm round glass coverslips (2 × 10^5^ cells/well, 6 well plates) 24 h after siRNA transfection. Ca^2+^ measurement was performed 72 h later. For cytosolic Ca^2+^ measurements, cells were incubated with 1 μM of Fura-2/AM (Invitrogen) in Krebs-HEPES buffer (11.5 mM HEPES, 120 mM NaCl, 6 mM KCl, 1.8 mM CaCl_2_, 1.2 mM MgCl_2_, 10 mM D-glucose, pH 7.6) for 1h15 at room temperature. Coverslips were then wash promptly and mounted in a heated (37 °C) microscope chamber. Cells were alternately excited at 340 and 380 nm using a Lambda DG-4 Ultra High Speed Wavelength Switcher (Sutter Instrument, Novato, CA, USA) coupled to an Axiovert 200M inverted microscope (Zeiss, Zaventem, Belgium). Images were acquired with a Zeiss Axiocam camera coupled to a 510 nm emission filter and analysed with Axiovision software (Zeiss). For mitochondrial Ca^2+^ measurements, cells were incubated 30 min at room temperature in Krebs-HEPES buffer containing 5 µM Rhod-2/AM and rinsed 30 min in Krebs-HEPES buffer. Cells were excited at 545 nm and images were acquired at 590 nm.

### 4.5. Cytofluorometric Assessment of Apoptosis-Associated Parameters and ROS Production

72 h after siRNA transfection, A549 cells were treated with CDDP. 24 h later, they were harvested and stained with propidium iodide (PI, 1 μg/mL, Sigma-Aldrich) for plasma membrane breakdown, 3,3′dihexiloxalocarbocyanine iodide (DiOC_6_(3), 40 nM, Molecular Probes—Fisher Scientific, Fisher Scientific, Erembodegem, Belgium) for mitochondrial transmembrane potential (ΔΨm) dissipation, H2DCFDA (5 μM, Molecular Probes—Fisher Scientific) for reactive oxygen species (ROS) generation. Only intact (PI-) cells were analyzed for ROS production. Cytofluorometric analyses were performed on a FACSCalibur flow cytometer equipped with Cell Quest Pro software (Becton Dickinson, Erembodegem, Belgium).

### 4.6. Statistical Analysis

Data are presented as means ± SD. Student’s *t*-test was used to determine statistical significance.

## 5. Conclusions

Taken together, our data revealed that SOCE triggers an increase in mitochondrial Ca^2+^ concentration, which was dramatically reduced after STIM1 depletion. STIM1 depletion also decreased production of ROS evoked by CDDP treatment. This was accompanied by a diminution of the DDR and subsequent apoptosis. SOCE might therefore constitute a mechanism of resistance against chemotherapy and targeting SOCE mediators could then restore sensitivity to cytotoxic drugs such as CDDP.
