# Correction: Gualdani, R.; et al. Store-Operated Calcium Entry Contributes to Cisplatin-Induced Cell Death in Non-Small Cell Lung Carcinoma. *Cancers* 2019, *11*, 430

**DOI:** 10.3390/cancers12082023

**Published:** 2020-07-23

**Authors:** Roberta Gualdani, Marie de Clippele, Ikram Ratbi, Philippe Gailly, Nicolas Tajeddine

**Affiliations:** Laboratory of Cell Physiology, Institute of Neuroscience, Université Catholique de Louvain, 1200 Brussels, Belgium; roberta.gualdani@uclouvain.be (R.G.); marie.declippele@uclouvain.be (M.d.C.); ikram.ratbi@uclouvain.be (I.R.); philippe.gailly@uclouvain.be (P.G.)

The authors wish to make the following corrections to this paper [1]:

There was a mistake in the original version of the article in Figure 2. The right flow cytometry plot of Figure 2A (siTRPC1) does not correspond to the mentioned conditions, and is in fact the same as the left one (siCTRL). This is due to a mistake in the figure editing. However, the numbers on this plot are correct, as are the histograms quantifying the cytometry experiments shown in Figure 2B. The figure legend does not need to be changed.

Thus, the original Figure 2 listed below: 
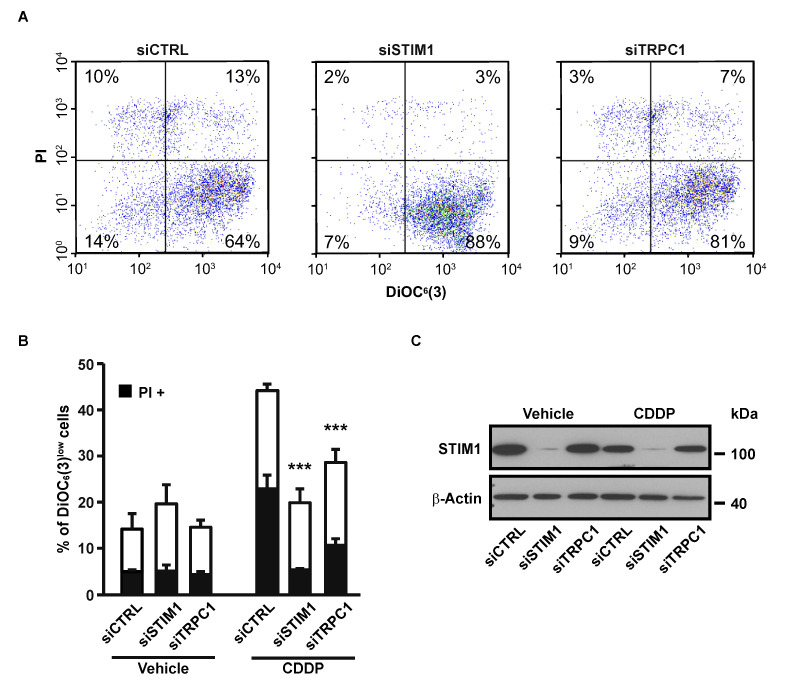
 should be replaced with the following version:

**Figure 2:**
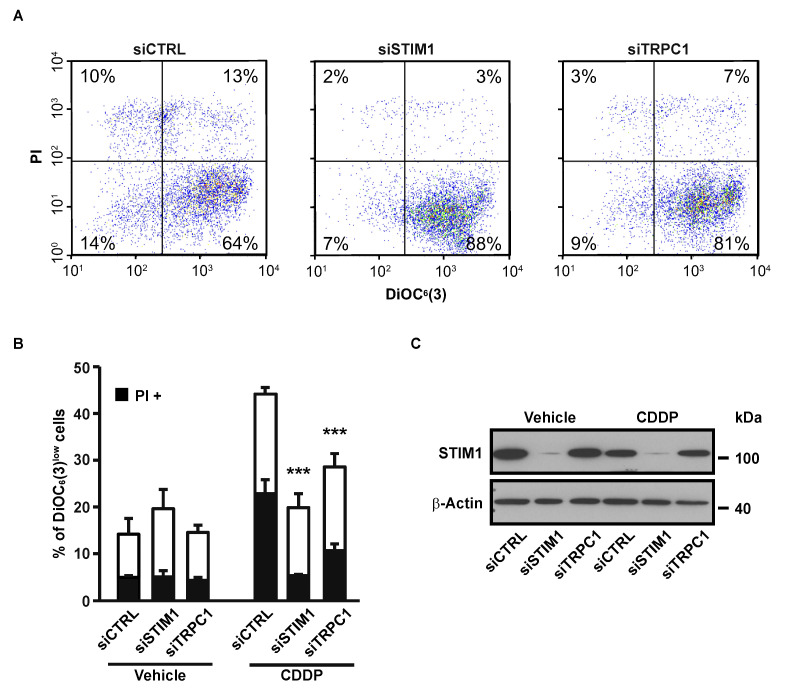
Effects of STIM1 and TRPC1 depletion on apoptosis-associated mitochondrial transmembrane potential (ΔΨ_m_) dissipation and plasma membrane permeabilization. (**A**) Following transient transfection (for 72 h) with either siRNA downregulating STIM1 (siSTIM1) or TRPC1 (siTRPC1) or with a control siRNA (siCTRL), A549 cells were left untreated or treated for additional 24 h with 25 µM CDDP, and labeled for the cytofluorometric assessment of ΔΨ_m_ [with the ΔΨ_m_-sensitive probe DiOC_6_(3)] and plasma membrane integrity (with propidium iodide, i.e., PI). (**B**) Quantification of data presented in A. White and grey columns represent the percentage of cells exhibiting ΔΨ_m_ loss alone [DiOC_6_(3)^low^] or in association with plasma membrane breakdown (PI^+^), respectively. Data are means of triplicate experiments ± S.D. and are representative of three independent experiments. Student’s *t* test was employed to assess statistical significance. *** *p* < 0.001. (**C**) Immunoblot analysis of STIM1 expression 96 h after transfection of A549 cells with siCTRL, siSTIM1 or siTRPC1. β-actin was used as loading control.

The authors state that this correction does not modify the scientific results of the study. The authors would like to apologize for any inconvenience caused by this mistake. 
